# Use of the Hospital Anxiety and Depression Scale in Patients with Oral Lichen Planus: An Institutional Experience

**DOI:** 10.7759/cureus.70521

**Published:** 2024-09-30

**Authors:** Dhanya M, Umamaheswari TN, Karthikeyan Ramalingam, Sowmya S

**Affiliations:** 1 Oral Medicine and Radiology, Saveetha Dental College and Hospitals, Saveetha Institute of Medical and Technical Sciences, Saveetha University, Chennai, IND; 2 Oral Pathology and Microbiology, Saveetha Dental College and Hospitals, Saveetha Institute of Medical and Technical Sciences, Saveetha University, Chennai, IND

**Keywords:** anxiety, depression, fear, hads, olp, oral lichen planus, prevalence, quality of life (qol), recurrence

## Abstract

Background

Oral lichen planus (OLP) is a chronic inflammatory disorder affecting the oral mucosa. It commonly appears in reticular and erosive forms, among other variants that include bullous, papular, and plaque types. This condition can greatly diminish a patient's quality of life due to prevailing discomfort and anxiety.

Materials and methodology

A retrospective study was conducted involving patients with OLP in the Department of Oral Medicine and Radiology, Saveetha Dental College and Hospitals, Chennai, India, measuring their anxiety and depression levels using the Hospital Anxiety and Depression Scale (HADS). Patient data were extracted from the private institution's electronic data recording system from June 1, 2023, to June 30, 2024. The retrieved data also included parameters such as age, gender, chief complaint, associated comorbidities, habit history, type of clinical variant, and previous medication history. SPSS Version 26.0 (IBM Corp., Armonk, NY) was employed for data analysis, utilizing Fisher's exact tests, chi-square tests, and Mann-Whitney U tests for comparisons, with a p-value of <0.05 considered as significant. To compare proportions between groups, the chi-square test was applied, and when the expected cell frequency was less than 5, Fisher’s exact test was used.

Results

Out of 1,500 outpatients in the department, 367 were diagnosed with OLP and 212 met the diagnostic criteria for inclusion in a one-year study (June 1, 2023, to June 30, 2024). The most frequently reported age group was 51-60 years, representing 27.4% (n = 58) of the total population, with a female predominance of around 62.7% (n = 133). Patients’ associated comorbidities revealed the prevalence of Grinspan syndrome in around 9.4% (n = 20). Additionally, the habit history indicated no significant history in 92% (n = 198) of cases. The majority of the study population was symptomatic, reporting a chief complaint of burning sensation (46.2%) (n = 98). The most common clinical variant reported was reticular (53.8%) (n = 114), followed by the erosive variant (36.8%) (n = 78). Medication history revealed that triamcinolone acetonide 0.1% (42.9%) (n = 91) was the most commonly prescribed medicine, followed by prednisolone 5 mg (32.5%) (n = 69). The HADS score indicated that 48.58% (n = 103) of patients had abnormal anxiety scores (mean of 15.8 ± 2.02), while 42.4% (n = 90) exhibited abnormal depression scores (mean of 16.3 ± 2.07). Notably, patients with the erosive variant showed higher anxiety scores compared to those with the reticular variant, with an average score of 9.3 ± 1.07, with a p-value of 0.041.

Conclusion

The findings underscore the substantial psychological burden associated with OLP, which affects patient’s emotional well-being and treatment adherence. Addressing these psychological concerns through regular assessments and supportive interventions is crucial for improving the standard of living.

## Introduction

Oral lichen planus (OLP) is a chronic inflammatory autoimmune disease that primarily affects the mucous membranes of the oral cavity [1]. Globally, OLP occurs at a frequency of 1.01%; Europe has the highest prevalence (1.43%), while India has the lowest (0.49%), with a cumulative malignant transformation rate of 0.2% [2,3]. There is currently no known cause for OLP, but it involves both antigen-specific and nonspecific inflammatory mechanisms. Additionally, stressors related to psychology are a major risk factor for the development of OLP. Consequently, managing OLP involves both psychological intervention and effective lesion therapy [4,5].

Clinically, OLP can manifest in several forms, showing a female predominance [6]. The common age distribution of OLP ranges from 30 to 50 years. Clinically, it is characterized by the presence of Wickham striae (white, lace-like patterns), desquamative gingivitis (inflammatory involvement of the gingiva with peeling), and a burning sensation, which often exacerbates while eating spicy or acidic foods [7].

Management of OLP often involves symptomatic treatment, among which topical and systemic corticosteroids are commonly used to reduce inflammation and alleviate symptoms [8]. Other treatment strategies in severe cases include immunomodulators, which require careful monitoring. Secondary management involves the use of analgesics and antiseptic mouthwashes to prevent secondary infections, as well as dietary modifications to avoid spicy foods [9]. A recent addition to the therapeutic management of OLP is photodynamic therapy, using particular photosensitizers such as methylene blue and 5-aminolevulinic acid with a definitive wavelength [10]. Additionally, platelet derivatives such as platelet-rich plasma and platelet-rich fibrin are also used nowadays [11]. Herbal formulations are currently widely used as alternatives for patients who are non-responsive to corticosteroids. Commonly used herbal agents include turmeric, aloe vera, tulsi, and green tea [12].

Recurrence of OLP is a common concern for patients and healthcare providers, with a reported rate of around 52.9% [13]. This chronic inflammatory disease may resolve temporarily but often recurs, leading to ongoing discomfort and anxiety [14]. Stress, immunological system dysfunction, and possible triggers such as specific drugs or dental materials are factors that can cause recurrence. The unpredictability of flare-ups can significantly impact a patient's quality of life, as they may experience pain, difficulty eating, and concerns about oral cancer. Effective management often involves regular monitoring, patient education, and tailored treatment strategies to minimize symptoms and reduce the likelihood of recurrence [15].

Several anxiety scales are used to measure anxiety levels in diverse populations, including the State-Trait Anxiety Inventory (STAI), Generalized Anxiety Disorder 7 (GAD-7), Beck Anxiety Inventory (BAI), Hamilton Anxiety Rating Scale (HAM-A), and Hospital Anxiety and Depression Scale (HADS) [16]. These scales provide valuable insights into a patient's anxiety levels, enabling healthcare providers to tailor interventions based on individual needs. Among the many anxiety scales available, the HADS is employed to assess patient's levels of anxiety and depression, featuring 14 items and a scoring range from 0 to 3 (Appendix). In the case of OLP, HADS can help identify the psychological impact of the condition, as patients often experience significant anxiety related to symptoms, potential complications, and the chronic nature of the disease [17,18].

The study aims to evaluate the presence of anxiety and depression in patients diagnosed with OLP using HADS. Additionally, the study's objective is to assess the levels of anxiety and depression among the various clinical variants of OLP.

## Materials and methods

Study characteristics

A retrospective study was performed in the Department of Oral Medicine and Radiology, Saveetha Dental College and Hospitals, Chennai, India, to evaluate anxiety and depression levels in patients with OLP using the HADS. The study received approval from the Saveetha Dental College Institutional Human Ethical Committee (Registration ID: IHEC/SDC/OMED-2202/23/211). Two researchers reviewed the case records of lichen planus patients from the electronic data recording device from June 1, 2023, to June 30, 2024, to ensure accurate research.

Selection of data

Patients aged 18 years and above presenting with OLP were included in the study. The study focused specifically on the adult population, who would have accurately self-reported psychological symptoms. Excluding lichenoid-like reactions and associated potentially malignant oral disorders, recurrent OLP lesions were included, ensuring that the study focused solely on new OLP lesions. Additionally, those under 18 years of age were excluded to avoid confounding factors that could impact the assessment of anxiety and depression levels. This helped maintain a homogenous study group, enhancing the validity of the findings on psychological impacts.

Patient assessment

Data were collected through an electronic data recording device, where information and scoring regarding the questionnaire were documented. The data containing the scoring was recorded by a structured questionnaire system, supplemented by interviews, which gathered detailed information on both psychological and physical symptoms. The questionnaire included questions on anxiety, depression, and general emotional well-being. Additionally, patients were assessed for extra-oral manifestations, such as skin or genital lesions. Based on the overall addition of scores from 14 questions, the HADS scale has been classified into three major domains: normal (0-7), borderline (8-10), and abnormal (11-21) [19]. In addition, comprehensive demographic and clinical information was recorded for each patient, including their age, gender, and main presenting complaints such as burning sensations and discoloration. Systemic comorbidities and associated habits, such as smoking, pan chewing, and gutkha use, were also noted. The six types of clinical variants documented were erosive, reticular, atrophic, bullous, plaque, and papular forms. This thorough method provided a complete view of each patient's condition, enabling a detailed analysis of how anxiety levels might be associated with specific demographic data.

Data analysis

The collected data were input into Excel (Microsoft Corp., Redmond, WA) and analyzed using SPSS Statistics software Version 26.0 (IBM Corp., Armonk, NY). Descriptive statistics were performed for basic parameters such as age, gender, and chief complaints. Calculations of categorical frequencies were made, and different data comparisons were performed using chi-square tests. Fisher's exact test was used when any expected cell frequency was less than 5, with a p-value of less than 0.05 deemed significant.

## Results

**Figure 1:**
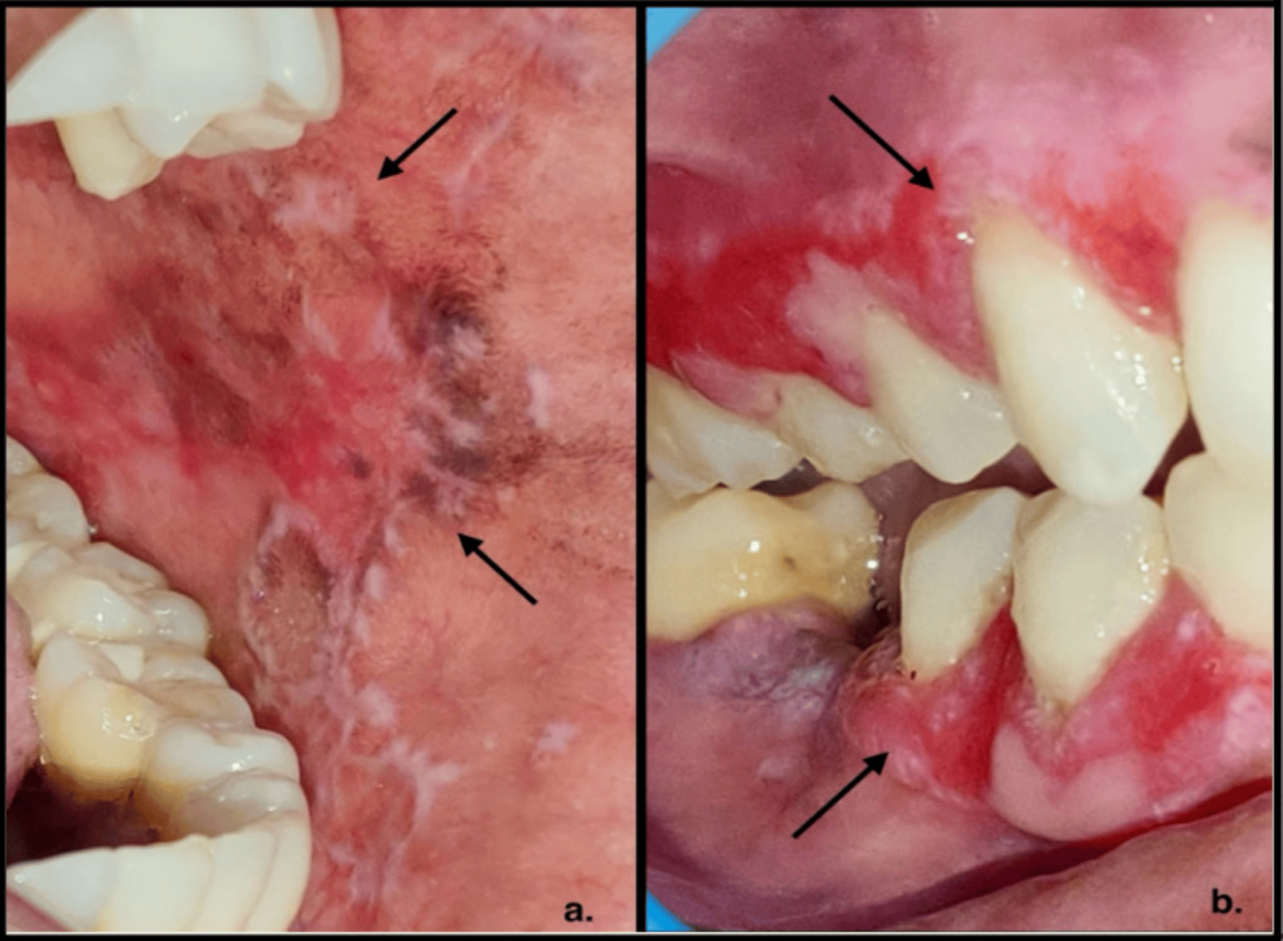
Clinical images of oral lichen planus Figure 1a with black arrows demonstrates the reticular variant of oral lichen planus, which appears as Wickham's striae, a distinctive white lace or net-like pattern, on the left buccal mucosa. Figure 1b with black arrows shows the erosive variant, presented as gingival desquamation.

Out of 1,500 outpatients in the Department of Oral Medicine and Radiology, 367 were diagnosed with OLP, representing 24.46% of the total outpatients (Figure 1). A total of 212 patients met the diagnostic requirements and were included in the study. Data were collected using the electronic data recording device from June 1, 2023, to June 30, 2024.

**Figure 2:**
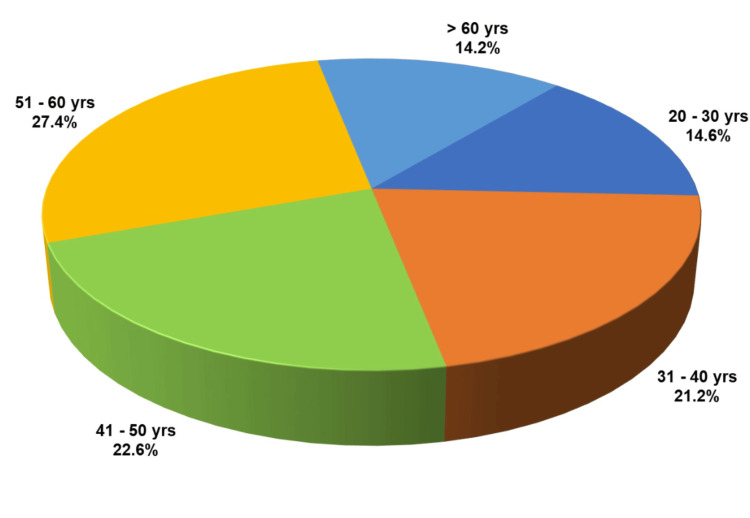
Age distribution of the study participants

**Figure 3:**
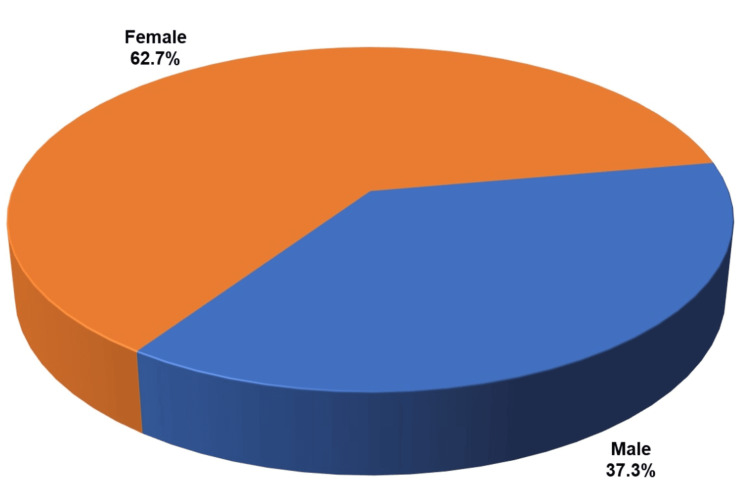
Gender distribution among study participants

Descriptive statistics were performed for parameters such as age, gender, habits, and clinical variants. For comparing proportions between groups (clinical variants + chief complaints, clinical variants + comorbidities, clinical variants + management, clinical variants + HADS) the chi-square test and Fisher’s exact tests were applied. For parameters with expected cell frequency of less than 5, Fisher’s exact test was used. The age groups of the patients were categorized into intervals of 10 years, with the most prevalent age group being 51-60 years (n = 58), accounting for 27.4% (Figure 2). A female predominance of around 62.7% (n = 133) was observed, compared to 37.3% (n = 79) for males (Figure 3).

**Figure 4:**
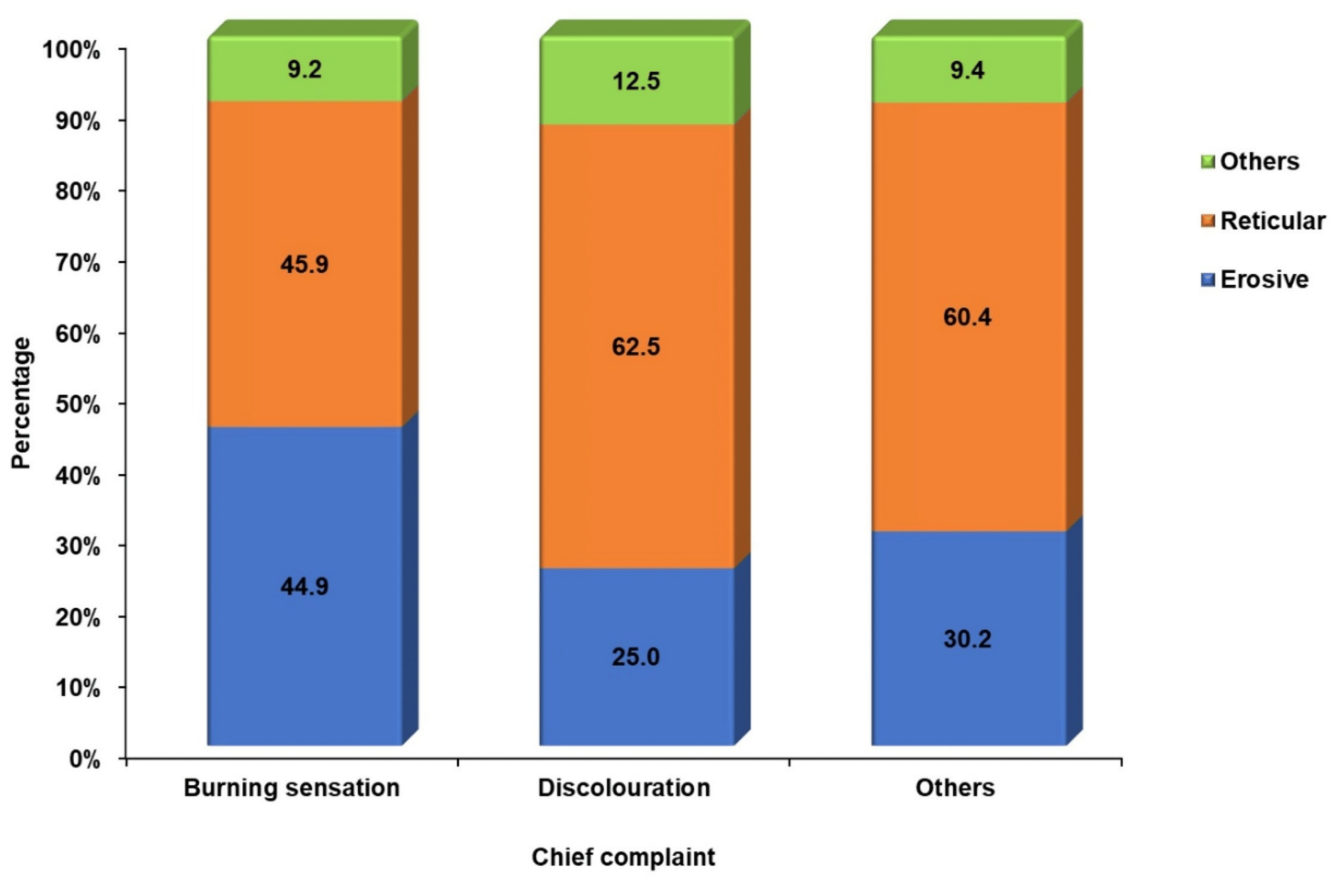
Clinical correlation with patient’s chief complaint

**Figure 5:**
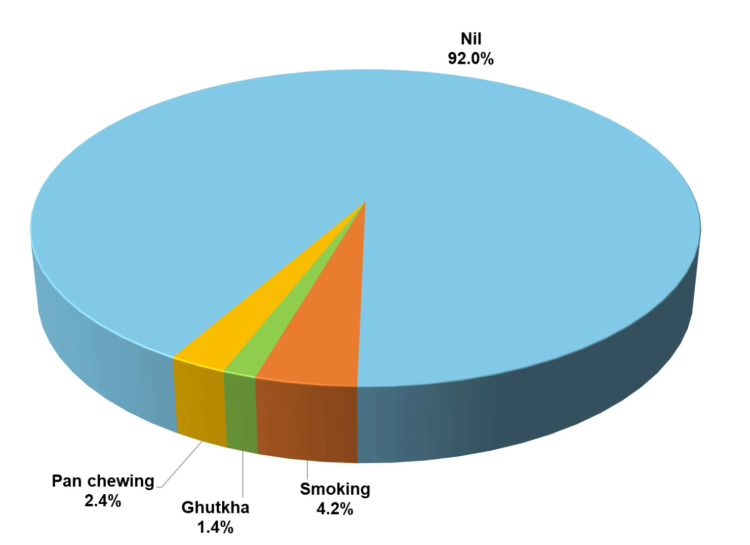
Habit associated with oral lichen planus

The patient’s chief complaints were assessed from the data, which included a burning sensation in 46.2% (n = 98), discoloration of the affected site in 3.7% (n = 8), and other dental complaints in 50% (n = 108). The correlation between the patient’s chief complaints and the clinical variants of OLP was also explored, revealing that the reticular variant of OLP was more symptomatic among the study participants with increased burning sensation, with a percentage of 45.9% (n = 45), while the erosive variant was at 44.9% (n = 44) (Figure 4). Patients associated with habits such as smoking, gutka, and pan chewing were also assessed, but this was found to be non-significant, as 92% (n = 198) of the study participants did not have these habits (Figure 5).

**Figure 6:**
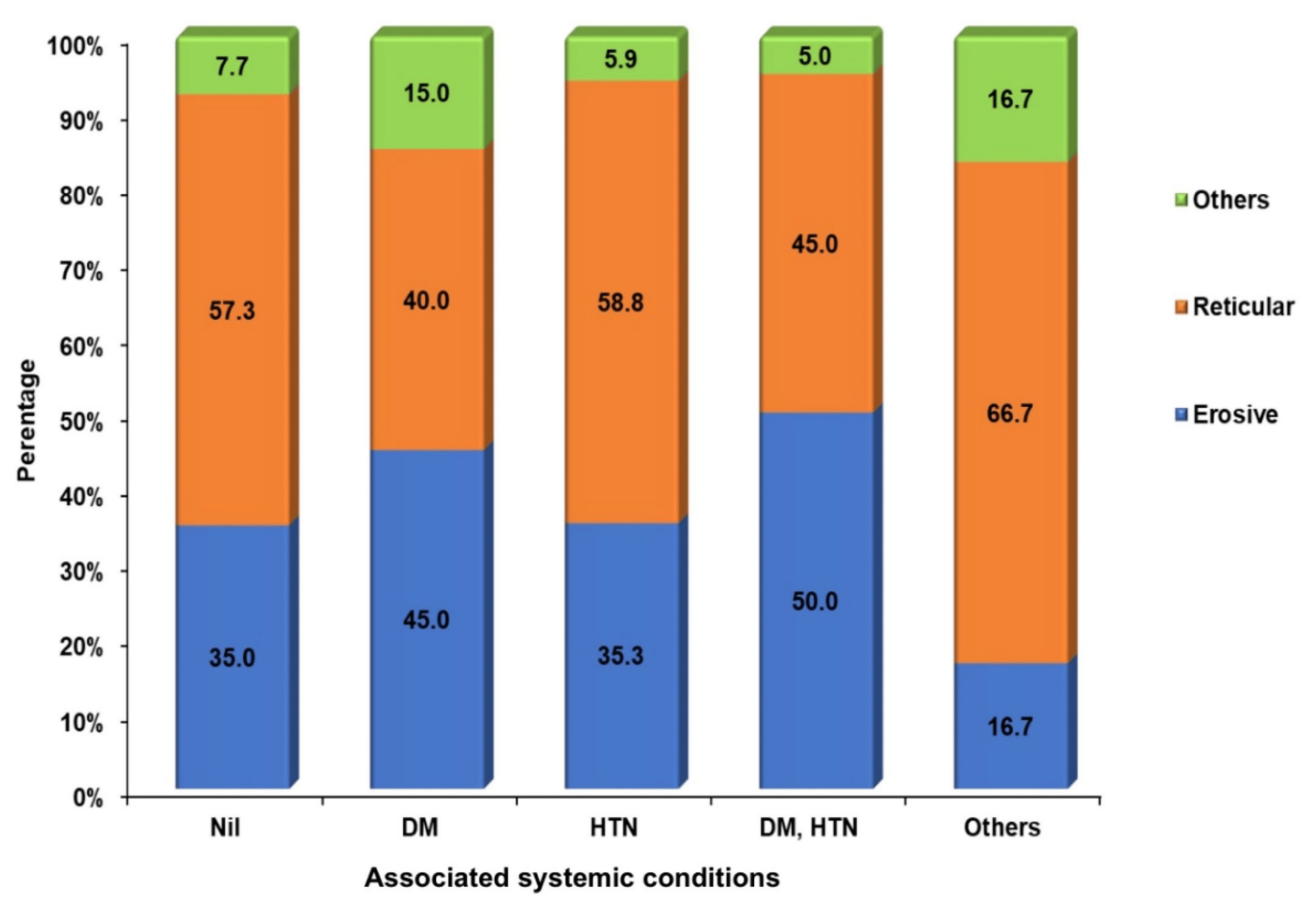
Associated systemic conditions and clinical variants of oral lichen planus Note: DM, HTN denoted group of patients presenting with both diabetes and hypertension (Grinspan syndrome) DM, diabetes mellitus; HTN, hypertension

**Figure 7:**
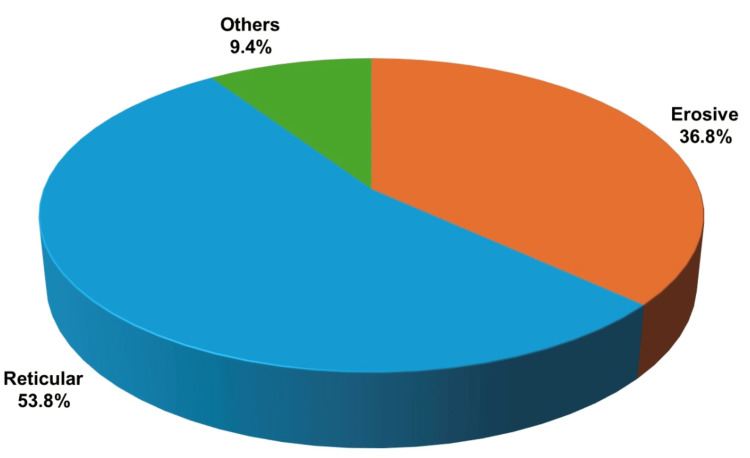
Distribution of clinical variants

The patient's comorbidities were assessed among the study participants, which included diabetes, hypertension, and a combination of diabetes and hypertension (Grinspan syndrome). The results indicated that 55.2% (n = 117) were free of systemic illness, 18.9% (n = 40) had diabetes, 8% (n = 17) had hypertension, and 9.4% (n = 20) had Grinspan syndrome. Additionally, 8.5% (n = 18) were categorized under "others," which included comorbidities such as epilepsy, coronary artery disease, respiratory disorders, and renal disorders. Correlations between comorbidities and clinical variants were observed, revealing that the erosive variant was associated with diabetes and hypertension in 45% (n = 18) and 35% (n = 6), respectively, while the reticular variant was associated with hypertension in 58.8% (n = 10) (Figure 6). Among the various forms of OLP, the reticular type showed the highest prevalence at 53.8% (n = 114), followed by the erosive variant at 36.8% (n = 78) (Figure 7).

**Figure 8:**
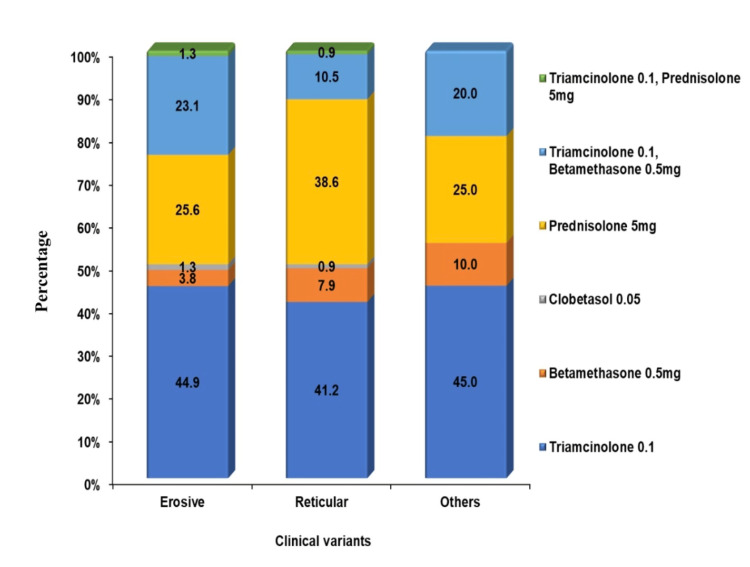
Distribution of various management administered for the reticular and erosive variants

The current assessment explored various medical management options for OLP. According to the data, the pharmacotherapy included triamcinolone acetonide 0.1%, betamethasone 0.5 mg, clobetasol 0.05%, and prednisolone 5 mg. Some data reported that combinations of steroid medications (triamcinolone acetonide 0.1% + betamethasone 0.5 mg, triamcinolone acetonide 0.1% + prednisolone 5 mg) were also prescribed. Among these, the most frequently prescribed drug was triamcinolone acetonide, at around 42.9% (n = 91), followed by prednisolone 5 mg, at around 32.5% (n = 69). A correlation of clinical variants and pharmacotherapy revealed that 44.9% (n = 35) of patients with the erosive variant and 41.2% (n = 47) of those with the reticular variant received triamcinolone acetonide 0.1% (Figure 8).

**Figure 9:**
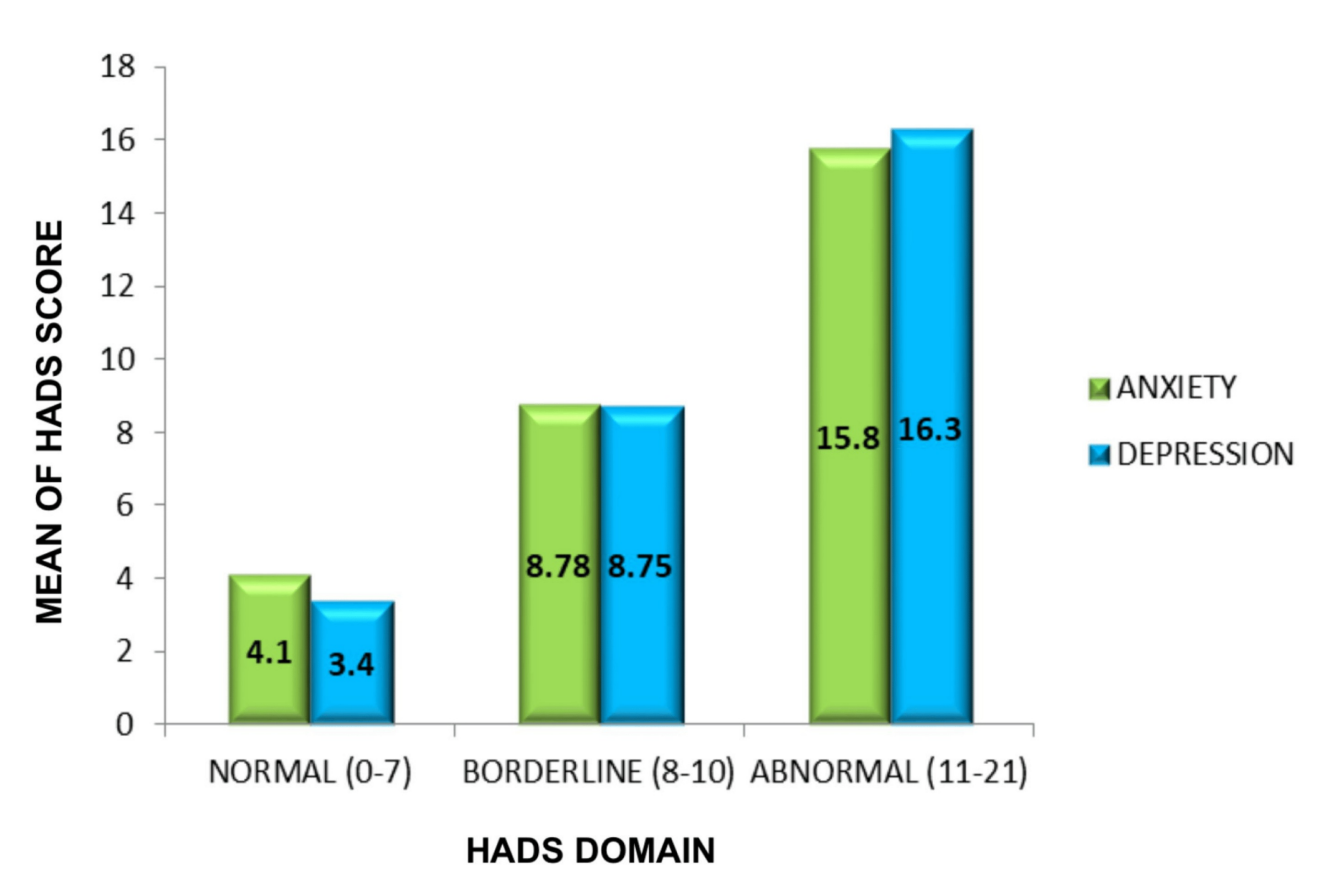
Distribution of HADS Figure 9 depicts the distribution of HADS domains along the X-axis (with a score of 0–7 as normal, 8–10 as borderline, and 11–21 as abnormal) and the mean scores observed on the Y-axis. HADS, Hospital Anxiety and Depression Scale

**Figure 10:**
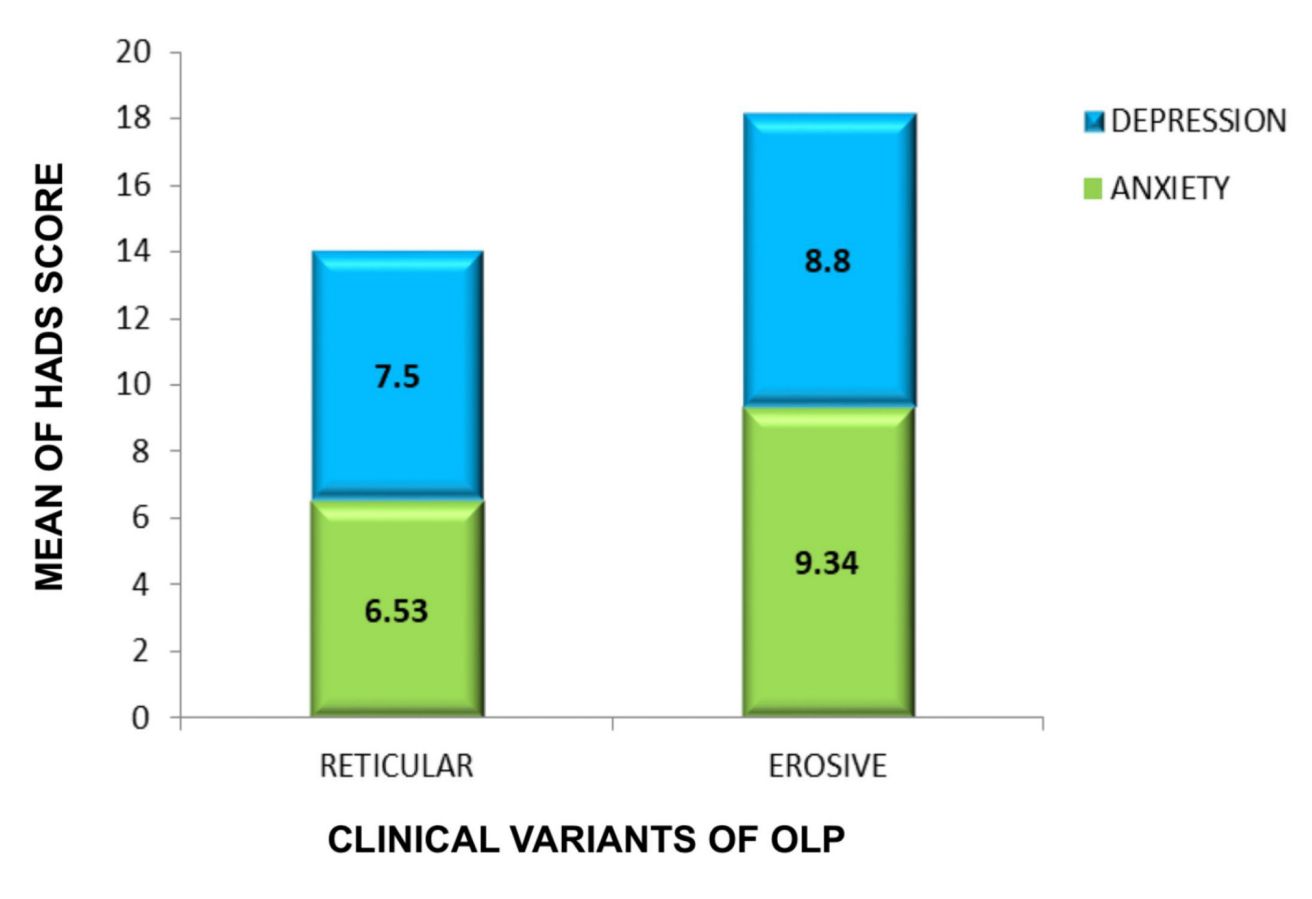
Distribution of HADS over the clinical variant HADS, Hospital Anxiety and Depression Scale; OLP, oral lichen planus

The anxiety and depression levels of patients were assessed using the HADS scale, which indicated that 15% (n = 32) (anxiety: mean 4.18 ± 1.7 standard deviation) and 18.39% (n = 39) (depression: mean 3.48 ± 1.33 standard deviation) of the population were considered normal based on a score range of 0-7. Additionally, 36.3% (n = 77) of participants with anxiety (mean 8.78 ± 0.74 standard deviation) and 41.98% (n = 83) with depression (mean 8.75 ± 0.78 standard deviation) were at a borderline level, with scores ranging from 8-10. Lastly, 48.58% (n = 108) of participants with anxiety (mean 15.87 ± 2.0 standard deviation) and 42.4% (n = 98) with depression (mean 16.3 ± 2.07 standard deviation) were at an abnormal level, with a higher level of anxiety indicated by scores ranging from 11 from 21. A significant p-value of 0.041 was observed (Figure 9). Additionally, the data revealed that those with the erosive form had a higher percentage of abnormal anxiety, at 59% (n = 61) (Mean 9.34 ± standard deviation), and depression, at 61% (n = 55) (Mean 8.8), whereas those with the reticular variant had abnormal anxiety, at 40.7% (n = 42) (mean 6.53 ± standard deviation), and depression, at 38% (n = 35) (mean 7.5 ± standard deviation) (Figure 10).

## Discussion

OLP has the potential to greatly affect the overall well-being of a patient. Psychological challenges faced by individuals can lead to heightened anxiety and distress. This discussion correlates our findings with existing literature to highlight the implications of anxiety and depression in OLP patients with HADS and compare our existing data with other anxiety scales.

Scully et al. reported a female predominance among the disease group, affecting people over 40 years of age [20]. These findings correlate with our current study, which observed a prevalent age distribution from 25 to 50 years and a female predominance of 62.7%. Similarly, in line with the current study, where the reticular variant was most prevalent at 53.8%, followed by the erosive variant at 36.8%, Sneha et al. demonstrated a prevalence of the reticular variant on the buccal mucosa to be 79% [21].

Research conducted by De Porras et al. and Kalkur et al. evaluated the overall anxiety levels in patients with OLP before undergoing therapy, revealing a moderate level of anxiety of around 47.5% compared to normal individuals [22,23]. This literature clearly shows that patients with OLP continue to experience inherent anxiety and depression. The current study proves that even after treatment completion, the fear persists in patients due to the impact of the lesions on day-to-day activities.

In addition to the HADS, other widely used assessment tools include the Beck Depression Inventory (BDI) and the Self-Rating Anxiety Scale (SAS), as utilized by Svanborg and Asberg and by Chen et al. [24,25]. The BDI is a 21-item questionnaire designed to assess the severity of depression, while the SAS focuses specifically on anxiety symptoms through self-reporting. These tools have been validated for use in various clinical settings, offering a comprehensive evaluation of emotional distress in patients. However, in this current study, we chose HADS for assessing anxiety and depression because of its simplicity and specificity in a hospital setting. HADS is a concise 14-item scale, divided equally between anxiety and depression, making it less time-consuming and easier to administer. It is particularly advantageous in medical conditions such as OLP, where the symptoms of anxiety and depression may overlap with somatic complaints. HADS was designed to minimize the influence of physical symptoms on psychological assessments, which is critical for patients with chronic illnesses. Furthermore, it has been extensively validated for use in various chronic conditions, making it an ideal tool for assessing psychological impacts in OLP patients.

Using HADS, this study was able to identify that 22% of patients with the erosive variant exhibited abnormal anxiety scores, suggesting that the psychological burden of OLP extends beyond localized concerns. HADS effectively captured this broader psychological response, highlighting its utility in chronic disease contexts.

The current study has certain limitations, including the limited duration of the study and the lack of comparison of the current data with previous data. Furthermore, the study did not account for potential confounding variables, such as other psychological conditions or life stressors, which could influence the reported anxiety and depression levels. Lastly, the lack of longitudinal follow-up means that the study cannot capture changes in psychological well-being over time, particularly in a chronic condition such as OLP. These limitations suggest the need for further research to enhance understanding of the psychological impacts of this condition.

The significant impact of OLP on patients is crucial for healthcare providers to implement strategies that address these psychological concerns. This could include regular psychological assessments, counselling, and educational interventions aimed at reducing anxiety and improving coping mechanisms. Moreover, fostering a supportive patient-provider relationship can help alleviate fears and encourage open discussions about concerns related to recurrence.

## Conclusions

Studies utilizing the HADS in OLP patients have shown that elevated anxiety and depression scores are common, reflecting the emotional burden associated with managing a chronic oral condition. By using the HADS, healthcare providers can better understand the mental health needs of OLP patients, facilitating more comprehensive care that addresses both physical symptoms and psychological well-being. This holistic approach can lead to a better future for patients.

## Figures and Tables

**Figure 11:**
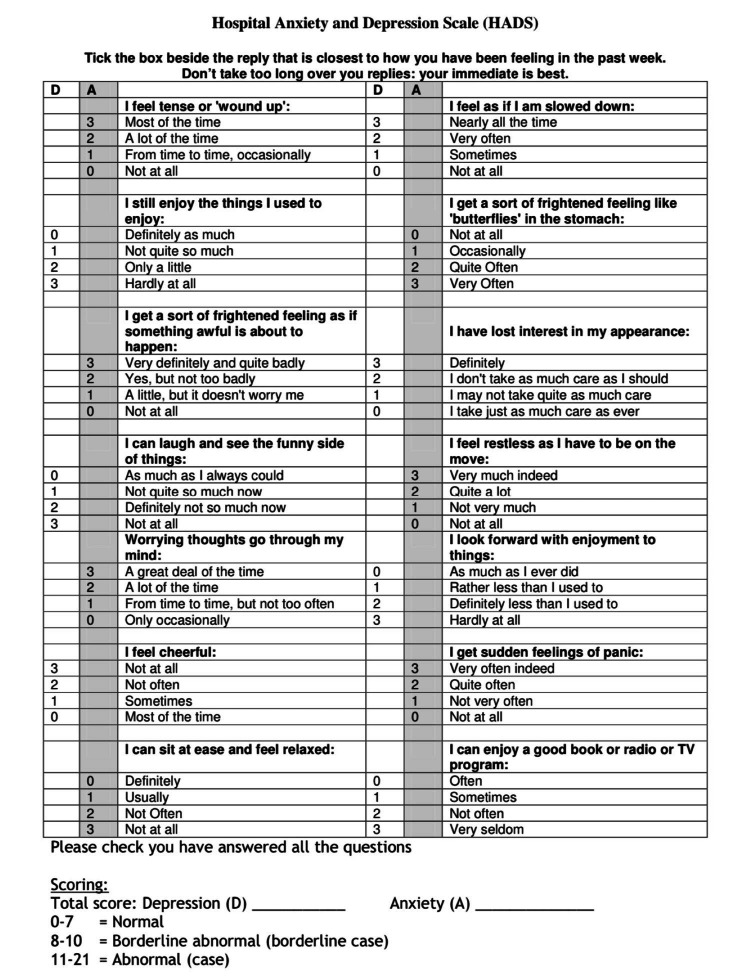
HADS questionnaire HADS, Hospital Anxiety and Depression Scale
